# Biclustering Models for Two-Mode Ordinal Data

**DOI:** 10.1007/s11336-016-9503-3

**Published:** 2016-06-21

**Authors:** Eleni Matechou, Ivy Liu, Daniel Fernández, Miguel Farias, Bergljot Gjelsvik

**Affiliations:** 1 School of Mathematics, Statistics and Actuarial Science, University of Kent, Cornwallis Building, Canterbury, CT2 7NF UK; 2 School of Mathematics and Statistics, Victoria University of Wellington, Wellington, New Zealand; 3 Centre for Research in Psychology, Behaviour, & Achievement, Coventry University, Coventry, UK; 4 Oxford Mindfulness Centre, University of Oxford, Oxford, UK; 5 Department of Psychology, University of Oslo, Oslo, Norway

**Keywords:** EM algorithm, fuzzy clustering, Likert scale, proportional odds

## Abstract

The work in this paper introduces finite mixture models that can be used to simultaneously cluster the rows and columns of two-mode ordinal categorical response data, such as those resulting from Likert scale responses. We use the popular proportional odds parameterisation and propose models which provide insights into major patterns in the data. Model-fitting is performed using the EM algorithm, and a fuzzy allocation of rows and columns to corresponding clusters is obtained. The clustering ability of the models is evaluated in a simulation study and demonstrated using two real data sets.

**Electronic supplementary material:**

The online version of this article (doi:10.1007/s11336-016-9503-3) contains supplementary material, which is available to authorized users.

## Introduction

Measurement data with ordinal categories occur frequently and in many fields of application. For example in medicine, a continuous clinical response is often categorised into ordered subtypes based on histological or morphological terms. In a questionnaire, Likert scale responses might be “better”, “unchanged” or “worse”. When analysing such data, it is of interest to link the ordinal responses to a set of explanatory variables.

Despite being introduced more than 3 decades ago, the proportional odds model (PO, McCullagh, 1980) is still frequently employed in analysing ordinal response data in, for example, agriculture (Lanfranchi, Giannetto, & Zirilli, 2014), medicine (Skolnick et al., 2014; Tefera & Sharma, 2015) and socioeconomic studies (Pechey, Monsivais, Ng, & Marteau, 2015).

One motivation for the PO model assumes that the ordinal response has an underlying continuous variable (Anderson & Philips, 1981), called a latent variable, that follows a logistic distribution. The extensive use of the PO model is due to its parsimony for modelling the effect of covariates on the response, compared to other similar models such as the baseline-category logit model, thanks to the use of the proportional odds property (Agresti, 2010, Sect. 3.3.1). Additionally, the model parameters are invariant to the way the categories for the ordinal response are formed (Agresti, 2010, Sect. 3.3.3).

In the analysis of two-mode data matrices, with the modes being for example subjects and questions and with all of the elements being ordered categorical responses, one might be interested in modelling the effect of both the rows and columns on the response. An example of such data is an *n* by *p* matrix that summarises the responses of *n* individuals to *p* questions, each with *q* possible (ordered) responses. In this case, the PO model can be fitted to identify, for example, individuals and questions that tend to be linked with higher values of the ordinal response.

However, the number of parameters in the PO model increases as the number of rows or columns in the data set increases. As a result, interpretation becomes problematic for large data sets. Identifying patterns related to the heterogeneity of the data, for example clusters of rows or columns that have similar effect on the response, is challenging. Therefore, the formulation of model approaches taking into account the row and column cluster structure of the data is needed.

The work in this paper has been motivated by this need to model potential heterogeneity among the, assumed independent, ordinal responses in two-mode data by identifying row and/or column clusters. As well as a single-mode clustering, our proposed model provides a two-mode clustering, or biclustering, for fuzzy allocation of the rows and/or columns to corresponding clusters. This way, the number of parameters can be reduced considerably as rows and/or columns are clustered in corresponding homogeneous groups assumed to have the same effect on the response. The results provide insights into major patterns in the data, and row/column clusters can be compared and ranked according to their effect on the ordinal response.

A number of model-based or distance-minimising biclustering methods exist that allocate, probabilistically or not, the rows and columns of a data set containing continuous, binary or count data to corresponding clusters. Examples include the double *k*-means method of Vichi (2001) and Rocci and Vichi (2008) which, as the name suggests, resembles the *k*-means algorithm (Hartigan & Wong, 1979), and the block mixture models of Govaertand and Nadif (2003, 2010). Pledger and Arnold (2014) have recently proposed a group of likelihood-based models fitted using the Expectation–Maximisation algorithm (EM) (Dempster, Laird, & Rubin, 1977) for simultaneous fuzzy clustering of the rows and columns of binary or count data.

The cluster analysis given by Pledger and Arnold (2014) can be considered as a multivariate approach using latent modelling. For both ordered and unordered categorical variables, Desantis, Houseman, Coull, Stemmet-Rachamimiv, and Betensky (2008) proposed a one-mode clustering method based on latent modelling, which has been widely applied in many fields (e.g. Desantis, Andrés Houseman, Coull, Nutt, & Betensky, 2012; Eluru, Bagheri, & Miranda-Moreno, 2012; Molitor, Papathomas, Jerrett, & Richardson, 2010; Scharoun-Lee et al., 2011).

In this paper, we generalise the Pledger and Arnold (2014) work to the case of ordinal categorical response data, specifically using the PO model parameterisation. The proposed model structure is an extension of the one-mode clustering model given by Desantis et al. (2008).

Section 2 describes the model structure. The performance of several model selection criteria in selecting the true number of clusters in the data when our proposed model is used is assessed in Sect. 3.1. The reliability of the clustering resulting from our proposed model is evaluated, using simulation, in Sect. 3.2. Finally, applications to two real data sets are shown in Sects. 4.1 and 4.2 and the resulting clusters are compared to those obtained by double *k*-means (Vichi, 2001).

## Materials and Methods

### Background: Proportional Odds Model

Consider the data set as an $n\times p$ matrix $\mathbf Y$ with entry $y_{ij}$ the realisation of a categorical distribution with *q* cells and $\theta _{ij1},\ldots ,\theta _{ijq}$ probabilities, $\sum _{k=1}^{q}\theta _{ijk}=1, \forall i, j$. Let the set of model parameters be denoted by $\pmb \phi$.

Under the PO model, and in the case where the additive effect of rows and columns on the response is considered

1 $$\begin{aligned} \theta _{ijk}=\left\{ \begin{array}{ll} \frac{\exp (\mu _{k}-\alpha _i-\beta _j)}{1+\exp (\mu _{k}-\alpha _i-\beta _j)}, &{} \quad k=1\\ &{} \\ \frac{\exp (\mu _{k}-\alpha _i-\beta _j)}{1+\exp (\mu _{k}-\alpha _i-\beta _j)}-\frac{\exp (\mu _{k-1}-\alpha _i-\beta _j)}{1+\exp (\mu _{k-1}-\alpha _i-\beta _j)}, &{}\quad 1< k < q\\ &{} \\ 1-\sum _{k=1}^{q-1}\theta _{ijk}, &{}\quad k=q\\ \end{array}\right. \end{aligned}$$

or alternatively,

2 $$\begin{aligned} \text{ logit }\left[ P(Y_{ij}\le k)\right] =\left\{ \begin{array}{ll} \mu _{k}-\alpha _i-\beta _j,&{} \quad 1\le k<q\\ +\infty , &{} \quad k=q,\\ \end{array}\right. \end{aligned}$$

where $\mu _{k}$ is the *k*th cut-off point, with $\mu _1<\mu _2<\cdots <\mu _{q-1}$, and $\alpha _i$, $\beta _j$ are, respectively, the effect of row *i*, column *j* on the response, with $\alpha _1=\beta _1=0$. The total number of model parameters is equal to $\nu =(q - 1) + (n-1) + (p-1)$.

### Biclustering: Simultaneous Clustering of Rows and Columns

Suppose that the rows come from a finite mixture with *R* components or row clusters while the columns come from a finite mixture with *C* components or column clusters. Rows that belong to the same row cluster, *r*, are assumed to have the same effect on the response, modelled using parameter $\alpha _r$. Similarly, columns that belong to the same column cluster c have the same effect on the response modelled by parameter $\beta _{c}$. If cell *i*, *j* belongs to row group *r* and column group *c* then, under the PO model and assuming an additive effect of the clusters on the response,

3 $$\begin{aligned} \text{ logit }\left[ P(Y_{ij}\le k)\right] = \mu _{k}-\alpha _r-\beta _{c}\; \text {if}\; 1\le k<q\; \text {and}\;+ \infty \; \text {otherwise}. \end{aligned}$$

The proportion of rows in row group *r* is $\pi _r$ and the proportion of columns in column group *c* is $\kappa _{c}$, with $\sum _{r=1}^R{\pi }_r=\sum _{c=1}^C{\kappa }_{c}=1$. As the rows and columns in the same row and column cluster, respectively, share the same parameters, $\alpha _r$ and $\beta _{c}$, respectively, there are now $(q-1)+2(R-1)+2(C-1)$ parameters in the model, where $R\le n$ and $C \le p$. Choosing $R \ll n$ and $C \ll p$ ensures that the number of independent parameters in this model is lower than the number of parameters in the proportional odds model formulated in expression (2).

However, cluster membership is typically unknown and hence the (incomplete data) likelihood sums over all possible partitions of rows into *R* clusters and over all possible partitions of columns into *C* clusters

4 $$\begin{aligned} \ell ({{\pmb \phi }}, {\pmb \pi },{\pmb \kappa } |\mathbf {Y})=\log \left[ \sum _{c_{1}=1}^{C} \cdots \sum _{c_{p}=1}^{C} \kappa _{c_{1}} \cdots \kappa _{c_{p}} \sum _{r_{1}=1}^{R} \cdots \sum _{r_{n}=1}^{R} \pi _{r_{1}} \cdots \pi _{r_{n}} \prod _{i=1}^{n}\prod _{j=1}^{p}\prod _{k=1}^{q}{{\theta }_{r_ic_jk}^{I(y_{ij}=k)}} \right] , \end{aligned}$$

where $\pi _{r_i}$ and $\kappa _{c_j}$ is the proportion of rows and columns, respectively, that belong to row group *r*, column group *c* for the particular partition *i*, *j*, of rows and columns into *R* and *C* clusters, respectively.

Here, following Pledger and Arnold (2014, Sect. 2.2.2), we adopt a finite mixture model which, assuming row-based conditional independence, we can describe using the following (incomplete data) log-likelihood

5 $$\begin{aligned} \ell ({{\pmb \phi }}, {\pmb \pi },{\pmb \kappa } |\mathbf {Y})=\log \left[ \sum _{c_1=1}^C\ldots \sum _{c_{p}=1}^C {\kappa }_{c_1}\ldots {\kappa }_{c_{p}}\prod _{i=1}^n \left\{ \sum _{r=1}^R\pi _r\prod _{j=1}^p\prod _{k=1}^q{\theta }_{rc_jk}^{I(y_{ij}=k)}\right\} \right] , \end{aligned}$$

which sums over the possible column cluster partitions only. Equation (5) is obtained from Eq. (4) by taking terms of the *i* product through the *r* sums.

The additive model shown in Eq. (3) can be extended to a model which allows for an interaction between the row and column cluster effects, denoted by parameters $\gamma$, by modelling the logits of the cumulative probabilities as

6 $$\begin{aligned} \text{ logit }\left[ P(Y_{ij}\le k)\right] =\mu _{k}-\alpha _r-\beta _{c}-\gamma _{rc}\; \text {if}\; 1\le k<q\; \text {and}\;+\infty \;\text {otherwise}, \end{aligned}$$

and, assuming constraints $\sum _r\gamma _{rc}=0 \; \forall c$ and $\sum _{c}\gamma _{rc}=0 \; \forall r$, increasing the number of parameters by $(R-1)(C-1)$ compared to the additive case.

The model can also be altered to consider one-mode clustering, and the set of different models that can be fitted are shown in Table 1 with details given in Appendix A. The first two columns in Table 1, labelled as “*R*” and “*C*”, denote, respectively, the number of row and column clusters assumed in the model when $R=1$ and $C=1$ all rows/columns are homogeneous forming a single row/column cluster, when $R=n$ and $C=p$ all rows/columns are heterogeneous, each forming its own row/column cluster, when $R=r$ and $C=c$ there are *r* and *c* homogeneous row/column clusters, respectively. Additionally, models incorporating an interaction term are indicated by the associated parameters $\gamma _{lk}$ with *l* indexing the row clusters and *k* the column clusters.

**Table 1:** Model set with corresponding number of parameters $\nu .$

*R*	*C*	$\text{ Logit }\left[ P(Y_{ij}\le k)\right] , \quad 1\le k<q$	$\nu$
*r*	1	$\mu _k-\alpha _r$	$(q-1)+2R-2$
*r*	*p*	$\mu _k-\alpha _r-\beta _j$	$(q-1)+2R+p-3$
*r*	*p*	$\mu _k-\alpha _r-\beta _j-\gamma _{rj}$	$(q-1)+Rp+R-2$
1	*c*	$\mu _k-\beta _{c}$	$(q-1)+2C-2$
*n*	*c*	$\mu _k-\alpha _i-\beta _{c}$	$(q-1)+2C+n-3$
*n*	*c*	$\mu _k-\alpha _i-\beta _{c}-\gamma _{ic}$	$(q-1)+Cn+C-2$
*r*	*c*	$\mu _k-\alpha _r-\beta _{c}$	$(q-1)+2R+2C-4$
*r*	*c*	$\mu _k-\alpha _r-\beta _{c}-\gamma _{rc}$	$(q-1)+RC+R+C-3$

The following constraints are placed, where appropriate: $\alpha _1=0,\; \beta _1=0,\; \sum _k\gamma _{kl}=0,\; \forall l,\; \sum _l\gamma _{kl}=0,\; \forall k,\; \sum _{r=1}^R\pi _r=1,\; \sum _{c=1}^C\kappa _{c}=1$. $R=1$: a single row cluster, $R=r$: *r* row clusters, $R=n$: each row is in its own cluster. Similarly, $C=1$: a single column cluster, $C=c$: *c* column clusters and $C=p$: each column is in its own cluster. For example, when $R=1$, $C=c$, the rows form one cluster, while the columns form *c* clusters and the logits of the cumulative probabilities in the PO model for column cluster *c* and $1\le k<q$ are $\text{ logit }\left[ P(Y_{ij}\le k)\right] =\mu _{k}-\beta _{c}$, for all rows. If on the other hand $R=n$, $C=c$, the cumulative probabilities for row *i*, column cluster *c* are, assuming an interaction between row and column effects and $1\le k<q$, $\text{ logit }\left[ P(Y_{ij}\le k)\right] =\mu _{k}-\alpha _i-\beta _{c}-\gamma _{ic}.$

We denote by $Z_{ir}$ and $X_{jc}$ the indicator random variables for group membership of row *i* in row group *r* and column *j* in column group *c*, respectively. We use the EM algorithm (Dempster et al., 1977) by treating cluster membership as the missing data and derive estimates of the posterior probability of allocation of row *i* to row cluster *r* and of column *j* to column cluster *c*, given respectively by $E(Z_{ij})=\widehat{z}_{ir}$ and $E(X_{jc})=\widehat{x}_{jc}$, for $i=1,\ldots ,n$, $j=1,\ldots ,p$, $r=1,\ldots ,R$ and $c=1,\ldots ,C$ with $\sum _{r=1}^R\widehat{z}_{ir}=\sum _{c=1}^C\widehat{x}_{jc}=1,\ \forall i,j$.

The lack of a posteriori independence of the $Z_{ir}$ and $X_{jc}$ makes the evaluation of the expected value of their product computationally expensive as it requires a sum either over all possible allocations of rows to row groups, or over all possible allocations of columns to column groups. The variational approximation (Govaert & Nadif, 2005) which we employ (see Appendix A.3.1. for details) is a solution to this problem.

We give details of the EM algorithm steps in Appendix A for all models listed in Table 1.

All the computer code is written in **R** (R Core Team, 2014), and the (complete data) log-likelihood (given in Appendix A) is maximised using the Newton–Raphson algorithm provided as an option in *optim* to estimate parameters $\mu _1,\ldots ,\mu _{q-1}$ and the effects of row and column clusters, as well as their interaction, if these exist in the model being fitted. Since the likelihood surface is multimodal, the EM algorithm is started from a number of different points and the iteration with the highest obtained likelihood value is retained (Everitt, Landau, Leese, & Stahl, 2011). The **R** code to fit the models is available upon request from the first author.

## Simulation Studies

We have performed two simulation studies: one to evaluate the performance of 10 model selection criteria in recovering the true number of clusters when our proposed models are used (Sect. 3.1) and one to evaluate the reliability of our proposed models (Sect. 3.2).

### Model Selection

Since these are likelihood-based models, likelihood-based model selection criteria, such as AIC (Akaike, 1973), its small-sample modification ($\text{ AIC }_{c}$, Akaike, 1973; Burnham & Anderson, 2002; Hurvich & Tsai, 1989), BIC (Schwarz, 1978) and its Integrated Classification Likelihood version (ICL-BIC, Biernacki, Celeux, & Govaert, 2000), can be used to select amongst them.

Following Fernández, Arnold, and Pledger (2014), we set up a simulation study to empirically establish a relationship between our likelihood-based models for ordinal data, specifically using the PO model, and the performance of 10 information criteria (Table 2) in recovering the true number of cluster components.

**Table 2:** Information criteria summary table.

Criteria	Definition	Proposed for Depending on	
AIC (Akaike, 1973)	$-2\ell +2\nu$	Regression	$\nu$
$\text{ AIC }_{c}$ (Akaike, 1973)	$\text {AIC} + \frac{2\nu (\nu +1)}{np-\nu -1}$		$\nu$ and *np*
$\text{ AIC }_{u}$ (McQuarrie, Shumway, & Tsai, 1997)	$\text {AIC}_{c}+ np\log \left( \frac{np}{np-\nu -1}\right)$		
CAIC (Bozdogan, 1987)	$-2\ell +\nu (1+\log (\textit{np}))$		
BIC (Schwarz, 1978)	$-2\ell +\nu \log (\textit{np})$		
AIC3 (Bozdogan, 1994)	$-2\ell +3\nu$	Clustering	$\nu$
CLC (Biernacki & Govaert, 1997)	$-2\ell +2\text {EN}$		$\text {EN}$
NEC(R) (Biernacki, Celeux, & Govaert, 1999)	$\frac{\text {EN}}{\ell -\ell (1)}$		
ICL-BIC (Biernacki et al., 2000)	$-2\ell _{c}+\nu \log (\textit{np})$		$\nu$, $\textit{np}$ and $\text {EN}$
AWE (Banfield & Raftery, 1993)	$-2\ell _{c}+ 2\nu \left( \frac{3}{2} + \log (\textit{np})\right)$		

$\ell$ is the maximised incomplete-data log-likelihood (see Eq. 5); $\ell (1)$ is the maximised incomplete-data log-likelihood $\ell$ without clustering structure; and $\ell _{c}$ is the maximised complete-data log-likelihood given in Appendix A. The third column categorises the criteria according to whether they were proposed for model selection in a regression setting or for clustering. The last column indicates whether the penalty depends on the number of parameters, $\nu$, the total sample size which is the number of elements in the response matrix *Y*, *np*, and/or the entropy function, $\text {EN}(\cdot )=\ell - \ell _{c}.$

**Fig. 1:**
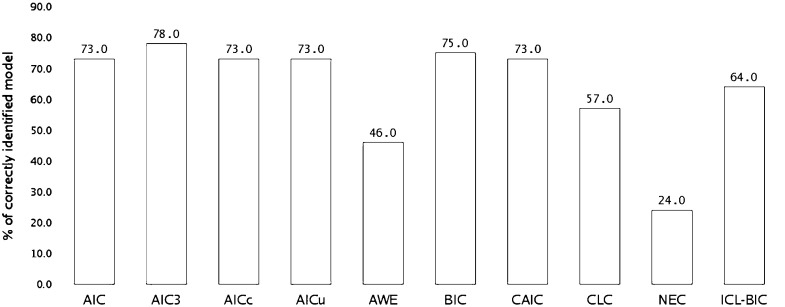
Simulation study to assess the performance of model selection criteria in recovering the true number of clusters for our proposed biclustering finite mixture PO (POFM) model. Bars depict the percentage of cases when the true model is correctly identified by each criterion, averaged across the five scenarios.

We set $n=150$, $p=15$, $q=4$, $R=3$ and $C=2$. We specified five scenarios by varying the row and column mixing proportions: a data set with similar dimensions ($n=150$ and $p=15$) to the data analysed in the example in Sect. 4.2 (Scenario 1), balanced row and column mixing proportions (Scenario 2), balanced column mixing proportions but unbalanced row proportions (Scenario 3), unbalanced row and column mixing proportions (Scenario 4) and one of the row mixing proportions close to zero (Scenario 5).

For each scenario, we simulated 100 data sets and noted the selected model using each of the 10 criteria out of models with $R=1,2,3,4,5$ and $C=1,2,3,4,5$. For each simulated data set, the EM algorithm was repeated 10 times with random starting points and the best ML estimates (those that led to highest log–likelihood value) were kept.

Figure 1 displays the percentage of cases in which each information criterion correctly recovered the true number of row and column clusters, i.e. the true model that generated the data, averaged across the five scenarios. AIC3 has the best performance (selecting the correct model in 78 % of cases), followed by BIC (75 %), AIC, $\text{ AIC }_{\text{ c }}$, $\text{ AIC }_{\text{ u }}$ and CAIC (73 %).

Our results are in accordance with Fonseca and Cardoso (2007) for the categorical case. ICL–BIC is underestimating the number of clusters (selecting a smaller number of clusters in 32 % of cases) and CLC is overestimating the number of clusters in 29 % of cases. A very poor performance is obtained by AWE and NEC (selecting the correct model in 46 and 24 % of cases, respectively).

It is important to highlight that these results are simply evaluating the ability of model selection criteria in selecting the right number of clusters in the mixture, but not necessarily in providing the best clustering structure for the data.

### Model Evaluation

In this section, we evaluate the performance of our proposed method in (i) biclustering, varying the cluster sizes and the sample size and (ii) one-dimensional row clustering, compared to that of double *k*-means (Vichi, 2001) and standard *k*-means, respectively.

(i) We set $R=3$, $C=2$ and $q=3$ or 5. The cutpoint values are obtained such that the response categories have equal probabilities for the baseline row and column cluster. That is, $P(Y_{ij}=1)=P(Y_{ij}=2)=\cdots =P(Y_{ij}=q)$ when row *i* belongs to the first row cluster and column *j* belongs to the first column cluster. The cutpoint values are $\{\mu _1=\log (1/2),\,\mu _2=\log (2)\}$ when $q=3$, and $\{\mu _1=\log (1/4),\,\mu _2=\log (2/3),\,\mu _3=\log (3/2),\,\mu _4=\log (4)\}$ when $q=5$. We consider $(\alpha _1,\,\alpha _2,\,\alpha _3)=(0,\,1,\,2)$, $(\beta _1,\,\beta _2)=(0,\,-1)$ and $\pi _1=\pi _2=\pi _3=1/3$. We vary *n*, *p*, *q* and $(\kappa _1,\,\kappa _2)$ as $n=(9,\,30,\,99)$, $p=(10,\,20,\,100)$, $q=(3,\,5)$ and $(\kappa _1,\,\kappa _2)=(0.5,\,0.5)$, $(0.4,\,0.6)$, $(0.3,\,0.7)$, $(0.2,\,0.8)$. The case with balanced column clusters assumes $(\kappa _1,\,\kappa _2)=(0.5,\,0.5)$. For an unbalanced case, the scenarios are from $(0.4,\,0.6)$ to $(0.2,\,0.8)$.

The response $\{Y_{ij}\}$ values are generated from a categorical distribution with size 1 and probabilities constrained as in expression (1). We assign the first 1/3 of rows to row cluster 1, the second 1/3 to row cluster 2 and the last 1/3 to row cluster 3. Similarly, the first $1/\kappa _1$ of columns are assigned to column cluster 1, and the rest of the columns to column cluster 2. We simulate 100 data sets for each scenario.

Table 3 shows the mean of parameter estimates obtained for $\alpha _2$, $\alpha _3$ and $\beta _2$ from 100 simulated data sets. We are aware of the bias in the estimated parameters when *n* or *p* are small. This is due to the fact that the clusters are not fixed and hence their effect on the response is not fixed either. For example, a group of subjects who belong to a certain cluster in the true model might be allocated into a different cluster for a simulated data set. Or, they might be separated into different clusters. However, when both *n* and *p* are large, the means are close to the true parameters, because it is less likely to allocate a large number of subjects to a wrong cluster and, hence, the clusters themselves are more similar to the true clusters.

**Table 3:** The average estimate obtained for each parameter over 100 simulations.

*n*	*p*	True	$(\kappa _1,\kappa _2)$							
			(0.5, 0.5)		(0.4, 0.6)		(0.3, 0.7)		(0.2, 0.8)	
			$q=3$	5	$q=3$	5	3	5	3	5
9	10	$\alpha _2=1$	1.40	1.46	1.43	1.58	1.43	1.56	1.46	1.49
	10	$\alpha _3=2$	3.03	1.99	2.30	2.22	2.40	1.95	2.37	1.99
	10	$-\beta _2=1$	1.33	1.02	0.98	0.90	0.76	0.86	0.73	0.71
	20	$\alpha _2=1$	1.42	1.38	1.42	1.43	1.41	1.38	1.45	1.40
	20	$\alpha _3=2$	1.88	1.91	1.95	1.90	2.07	1.84	2.00	1.92
	20	$-\beta _2=1$	0.95	0.91	1.43	0.84	1.14	0.93	0.71	0.69
	100	$\alpha _2=1$	1.31	1.42	1.34	1.43	1.38	1.44	1.37	1.44
	100	$\alpha _3=2$	1.88	1.97	1.90	2.00	1.92	1.99	1.92	2.00
	100	$-\beta _2=1$	1.07	0.88	0.93	0.81	1.24	1.02	0.98	0.88
30	10	$\alpha _2=1$	1.41	1.44	1.43	1.37	1.38	1.45	1.40	1.38
	10	$\alpha _3=2$	2.47	2.23	2.70	2.30	2.54	2.09	2.90	1.94
	10	$-\beta _2=1$	1.01	0.96	1.07	0.93	0.96	0.92	0.94	0.78
	20	$\alpha _2=1$	1.26	1.18	1.15	1.19	1.19	1.22	1.19	1.23
	20	$\alpha _3=2$	1.96	1.98	2.02	2.05	2.06	1.96	2.08	2.04
	20	$-\beta _2=1$	0.95	0.96	1.02	1.00	1.02	1.02	0.91	1.00
	100	$\alpha _2=1$	1.11	1.30	1.16	1.34	1.16	1.34	1.17	1.32
	100	$\alpha _3=2$	1.96	1.98	1.92	1.98	1.93	1.99	1.95	1.99
	100	$-\beta _2=1$	0.97	0.95	0.96	0.95	0.98	0.97	0.97	0.96
99	10	$\alpha _2=1$	1.22	1.24	1.42	1.31	1.22	1.22	1.39	1.19
	10	$\alpha _3=2$	2.28	2.16	2.32	2.22	2.33	2.21	2.47	2.16
	10	$-\beta _2=1$	1.00	0.97	1.01	0.99	1.01	1.00	0.96	0.98
	20	$\alpha _2=1$	1.05	1.02	1.03	1.03	1.06	1.01	1.06	1.06
	20	$\alpha _3=2$	2.04	1.99	2.04	2.04	2.05	1.97	2.06	2.01
	20	$-\beta _2=1$	1.01	0.99	1.00	1.00	0.98	0.99	0.99	0.98
	100	$\alpha _2=1$	1.03	1.13	1.04	1.14	1.05	1.19	1.04	1.17
	100	$\alpha _3=2$	1.99	1.99	1.99	2.00	1.97	2.00	1.99	1.99
	100	$-\beta _2=1$	0.99	1.00	1.00	0.99	1.00	0.99	1.00	0.99

**Table 4:** The average Rand index for 100 simulated data sets based on our proposed (POFM) and double *k*-means (dkm) methods.

*n*	*p*		$q=3$				$q=5$			
		$(\kappa _1,\kappa _2)=$	(0.5, 0.5)		(0.2, 0.8)		(0.5, 0.5)		(0.2, 0.8)	
		Cluster	POFM	dkm	POFM	dkm	POFM	dkm	POFM	dkm
9	10	Row	0.61	0.75	0.63	0.72	0.65	0.76	0.64	0.74
	10	Col.	0.64	0.63	0.60	0.54	0.65	0.59	0.59	0.52
	20	Row	0.74	0.78	0.73	0.80	0.75	0.76	0.71	0.78
	20	Col.	0.64	0.59	0.60	0.55	0.65	0.60	0.61	0.53
	100	Row	0.81	0.99	0.79	0.97	0.77	0.98	0.76	0.97
	100	Col.	0.66	0.62	0.62	0.55	0.70	0.64	0.65	0.57
30	10	Row	0.65	0.70	0.66	0.70	0.66	0.70	0.67	0.72
	10	Col.	0.75	0.76	0.80	0.60	0.86	0.75	0.73	0.67
	20	Row	0.76	0.77	0.78	0.78	0.78	0.79	0.78	0.80
	20	Col.	0.90	0.80	0.86	0.65	0.91	0.83	0.86	0.71
	100	Row	0.92	0.99	0.91	0.99	0.85	0.99	0.84	0.99
	100	Col.	0.91	0.84	0.93	0.74	0.93	0.87	0.94	0.79
99	10	Row	0.68	0.70	0.68	0.71	0.69	0.71	0.69	0.71
	10	Col.	0.99	0.96	0.95	0.85	0.99	0.97	0.93	0.88
	20	Row	0.78	0.80	0.80	0.81	0.82	0.81	0.81	0.82
	20	Col.	0.99	0.99	0.99	0.97	1.00	0.99	0.97	0.98
	100	Row	0.98	0.99	0.97	0.99	0.92	0.99	0.91	0.99
	100	Col.	0.99	0.99	1.00	0.98	1.00	0.99	1.00	0.99

Regardless of the bias, the overall result shows that for balanced cases with $(\kappa _1,\,\kappa _2)=(0.5,\,0.5)$, the estimates of the column effects are closer to the truth than for highly unbalanced cases $(\kappa _1,\,\kappa _2)=(0.2,\,0.8)$ when *n* is small. The unbalanced column clusters do not affect the quality of the row cluster effect estimates. In general, when both *n* and *p* increase, the quality of row cluster effect estimates improves. The standard errors are between 0.05 to 0.5 for the cases of $p=10$. For the other cases, they range from 0.001 to 0.08.

To evaluate the clustering ability of our proposed method, we calculate the average proportion of times that the pairwise grouping is correct (Rand index, Rand, 1971) over 100 simulated data sets. For example, if two rows are in the same cluster for the true model, but the proposed method allocates them to different clusters, then this pair is mis-clustered and vice-versa. We report the average Rand index for all row/column pairs in Table 4 when $(\kappa _1,\,\kappa _2)=(0.5,\,0.5)$ and $(0.2,\,0.8)$ for both our proposed approach and the double *k*-means algorithm (Vichi, 2001). The two approaches have similar performance which improves as *n* and *p* increase and when the column clusters are balanced. For our approach, the largest standard error is 0.03 for the highly unbalanced cases and most standard errors are between 0.001 to 0.01.

(ii) We set $R=3$ and $C=1$, i.e. $\text{ logit }\left[ P(Y_{ij}\le k)\right] =\mu _{k}-\alpha _r\; \text {if}\; 1\le k<q\; \text {and}\; +\infty \; \text {otherwise}$. The cutpoint values are calculated as in simulation setting (i) above. We vary *n* and *p* as $n=(9,\,30,\,99)$, $p=(10,\,20,\,100)$ and $\pi _1=\pi _2=\pi _3=1/3$ with $(\alpha _1,\,\alpha _2,\,\alpha _3)=(0,\,1,\,2),\,(0,\,2,\,4),\,(0,\,1,\,4)$ and $q=(3,\,5,\,7)$.

When *p* is large, there are more data points for each row. When *q* is large, the ordered categorical response has a finer scale. For the row cluster effects $\{\alpha _r,\,r=1,2,3\}$, the last setting $(0,\,1,\,4)$ gives an unbalanced effect where the difference between the first two clusters is small, but the first two clusters are quite different from the third cluster.

Table 5 shows the average Rand index for 1000 simulated data sets for each of the scenarios, comparing the proposed method (POFM) with *k*-means. All standard errors for the index are less than 0.0026. Most of them are around 0.001. POFM performs better than *k*-means when the cluster effects are balanced. In general, the greater *n*, *p*, *q* or the cluster effects are, the better the performance. The only case when *k*-means considerably outperforms POFM is when $(\alpha _1,\,\alpha _2,\,\alpha _3)=(0,\,1,\,4)$ and *p* is large. For this particular case, POFM fails to distinguish between Clusters 1 and 2, and partitions the individuals into only two clusters, leaving one of the clusters empty. However, the quality of the row clustering is still satisfactory, with the average Rand index greater than $70~\%$ in all cases.

**Table 5:** The average Rand index based on our proposed (POFM) and double *k*-means (dkm) methods for 1000 simulated data sets.

*n*	*p*	Method	$(\alpha _2,\alpha _3)=(1,2)$			$(\alpha _2,\alpha _3)=(2,4)$			$(\alpha _2,\alpha _3)=(1,4)$		
			$q=3$	5	7	3	5	7	3	5	7
9	10	POFM	0.61	0.63	0.64	0.73	0.78	0.80	0.74	0.75	0.75
		*k*-means	0.68	0.69	0.69	0.70	0.72	0.73	0.72	0.74	0.75
	20	POFM	0.70	0.72	0.73	0.79	0.86	0.88	0.77	0.76	0.75
		*k*-means	0.70	0.71	0.72	0.71	0.73	0.74	0.74	0.77	0.78
	100	POFM	0.85	0.84	0.83	0.94	0.94	0.86	0.75	0.75	0.75
		*k*-means	0.74	0.77	0.78	0.74	0.77	0.78	0.79	0.88	0.90
30	10	POFM	0.65	0.67	0.68	0.75	0.81	0.84	0.76	0.77	0.77
		*k*-means	0.66	0.67	0.68	0.70	0.72	0.73	0.71	0.74	0.76
	20	POFM	0.73	0.76	0.77	0.84	0.93	0.95	0.78	0.78	0.78
		*k*-means	0.70	0.72	0.72	0.72	0.75	0.76	0.75	0.80	0.81
	100	POFM	0.94	0.92	0.91	0.95	0.99	0.92	0.77	0.77	0.77
		*k*-means	0.79	0.83	0.86	0.76	0.84	0.87	0.93	0.97	0.98
99	10	POFM	0.67	0.68	0.69	0.76	0.84	0.88	0.76	0.77	0.78
		*k*-means	0.67	0.68	0.68	0.70	0.72	0.73	0.72	0.75	0.76
	20	POFM	0.75	0.78	0.80	0.86	0.95	0.97	0.79	0.78	0.78
		*k*-means	0.71	0.73	0.74	0.73	0.77	0.80	0.79	0.85	0.86
	100	POFM	0.98	0.97	0.96	0.97	1.00	0.97	0.78	0.78	0.78
		*k*-means	0.88	0.92	0.93	0.82	0.87	0.89	0.99	0.99	0.99

## Results: Case-Studies

### Religious beliefs

We consider part of the data set from a study first published by Wiech et al. (2008). Twelve individuals, self-classified as religious, replied to 16 questions, shown in Appendix B, all rated on a 6-point Likert scale, (1) “Strongly disagree”, ..., (6) “Strongly agree”. The questions were designed to assess an individual’s beliefs on the level of control that god (first 8 questions) and powerful other individuals (last eight questions) have on their lives.

The biclustering model proposed in Sect. 2 was fitted to the 12 by 16 matrix by considering $R, C = 2,\ldots ,4$. The model with the greatest support by AIC3 has $R=3$, $C=2$ and an interaction between row group effects and column group effects.

The two column clusters separate the questions into the two categories (god and others) almost perfectly. Cluster 1 includes questions {1, 2, 3, 4, 5, 6, 8, 10}, while Cluster 2 includes questions {7, 9, 11, 12, 13, 14, 15, 16}. The three row clusters are {3, 4, 5, 6, 8, 9, 10, 12}, {1, 2, 11} and {7}. Double *k*-means (Vichi, 2001) gives the same row clusters and similar column clusters {2, 3, 4, 5, 6, 8}, {1, 7, 9, 10, 11, 12, 13, 14, 15, 16}.

**Fig. 2:**
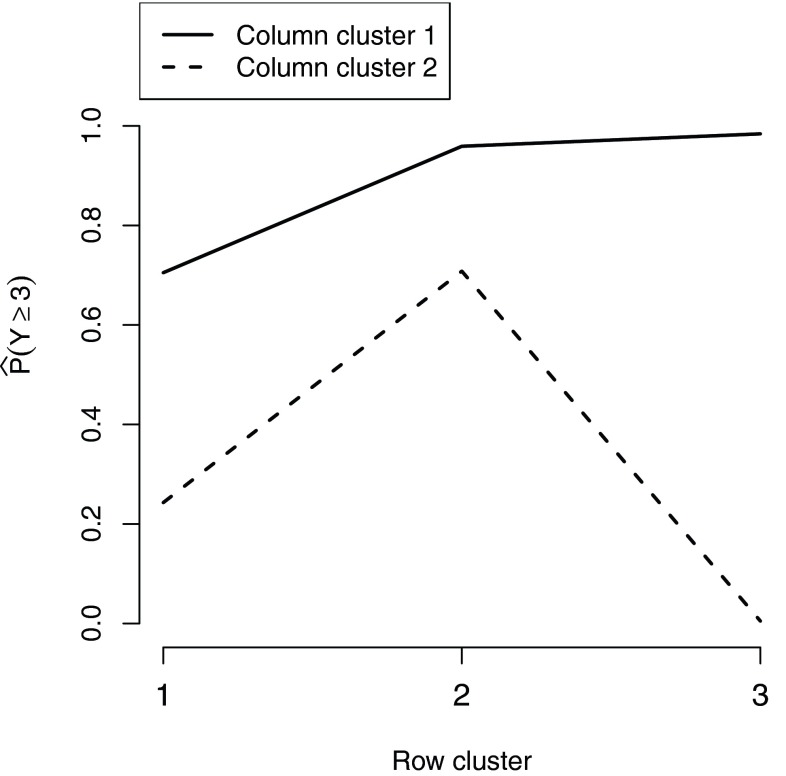
Estimated probabilities of replying 3 or above to each of the 2 column clusters for all 3 row clusters, as derived by the biclustering model with $R=3$, $C=2$.

The estimated probabilities of replying 3 or above to each of the two question clusters for the three row clusters are shown in Figure 2. All row groups tend to agree more with god-related questions than with questions related to the effect of other powerful people. The estimated probabilities of agreeing with the god-related questions do not vary considerably between the three row clusters. However, that is not the case for the second column group since Row Cluster 1 and particularly Row Cluster 3, which consists of Individual 7 alone, tend to give lower scores than individuals in Row Cluster 2. Note that in addition, Individual 7 strongly agrees with questions in Cluster 1, demonstrating more extreme views than individuals belonging to the other clusters, who tend to be more moderate in their answers.

### Attempted Suicides

The data set was collected as part of a study of patients admitted for deliberate self-harm (DSH) at the Acute Medical Departments of three major hospitals in Eastern Norway. We consider the answers of 151 individuals to 13 questions, shown in Appendix C, that were designed to assess the level of depression of the respondent by means of the Beck Depression Inventory-Short Form (BDI-SF) (Furlanetto, Mendlowicz, & Romildo Bueno, 2005). Response options range from 1 to 4, with higher scores indicating higher levels of depression (Beck, Schuyler, & Herman, 1974).

We fitted biclustering models with $R=2,\ldots ,5$ and *C*=2 or 3. The model supported by AIC3 has $R=5, C=2$ and an additive effect of row and column groups on the response.

The two column clusters are {1, 2, 3, 4, 5, 7, 8, 10, 13} and {6, 9, 11, 12}, with the first cluster receiving higher scores than the second ($\widehat{\beta }_2=-0.99 (0.10)$), suggesting that the nine questions of Cluster 1 are, possibly, markers of more severe forms of depression. The allocation of individuals to the five row groups is in proportion to 0.211, 0.266, 0.208, 0.030, 0.285. Double *k*-means (Vichi, 2001) gives the following column clusters: {2, 3, 4, 5, 6, 7, 8} and {1, 9, 10, 11, 12, 13}. For row clusters, we present the proportion of individuals from each of our clusters that are allocated to each of the double *k*-means clusters in Table 6, where it can be seen that with the exception of Cluster 4, the highest proportions appear in the diagonal of the table.

**Table 6:** Percent of individuals from the five POFM clusters, represented in the rows, that are clustered in the corresponding five double *k*-means (Vichi, 2001) clusters.

POFM cluster	Double *k*-means cluster				
	1	2	3	4	5
1	100	0	0	0	0
2	26	72	2	0	0
3	0	10	48	23	0
4	0	0	0	0	100
5	0	0	21	30	49

The fourth row cluster, which consists of four individuals, is believed to show the most signs of depression since $\widehat{\alpha }_4=1.8(0.32)$. The first cluster follows with $\widehat{\alpha }_1=0$ since it is the baseline, followed by Clusters 5 ($\widehat{\alpha }_5=-1.14 (0.12)$), 2 ($\widehat{\alpha }_2 -2.37(0.13)$), and 3 ($\widehat{\alpha }_3=-3.79 (0.16)$). In fact, no one in Cluster 4 contacted someone for help after their attempt, while the corresponding proportions for the other four clusters are all greater than $25~\%$, which demonstrates the greater determination of individuals in Cluster 4 to succeed in their attempt. Of course, the size of Cluster 4 is possibly too small to make meaningful comparisons of this type. However, the proportion of individuals in Clusters 1, 5, 2 and 3 that had at least one episode of DSH within three months after the study is, respectively, equal to 30, 24, 16 and $3.4~\%$. DSH is one of the most robust predictors of subsequent death by suicide (Hawton, Casanas, Comabella, Haw, & Saunders, 2013). The risk of suicide among DSH patients treated at hospital is 30- to 200-fold in the year following an episode compared to individuals with no history of DSH (Cooper et al., 2005; Hawton et al., 2012; Owens, Horrocks, & House, 2002). Our model has successfully ordered the groups in terms of their risk of DSH within three months since the data we considered were collected.

## Discussion

Our biclustering models identify homogeneous groups of both rows and columns in two-mode data sets of ordinal responses, reducing the number of parameters needed to adequately describe the data and therefore easing interpretation. They fully account for the ordinal nature of the responses, while, being likelihood-based, give access to tools for selecting between possible models.

We have performed an extensive simulation study to compare the performance of a number of model selection criteria in identifying the correct number of mixture components for models and data such as the ones we considered in our applications, conditional on using the EM algorithm and the variational approximation of Govaert and Nadif (2005). The variational approximation is known to produce local optima, and hence it is recommended to use different random starting values for several runs of the EM algorithm. Recently, Keribin, Brault, Celeux, and Govaert (2014) developed latent block models for categorical data, considering a Bayesian approach, which do not require the aforementioned approximation. The potential to develop such models for the PO parameterization is a matter of future research.

In the two real data applications considered, both including questionnaire-type data designed to gain knowledge about the participants’ personality, feelings and way of thinking, the clusters identified by the model agree with our knowledge of the system and provide useful insight of the characteristics of the participants. Especially in the example of Sect. 4.2, the way the participants were clustered agrees with information collected three months after the study was conducted.

In the analysis presented in Sect. 4.2 we have considered only individuals with complete records, excluding participants with missing data. Missing data are often present in similar studies; and, hence, future work could extend the models to deal with such issues. Fitting the models using a Bayesian approach could provide a way of dealing with the missing data and also of choosing the right number of clusters, as, for example, in van Dijk, van Rosmalen, and Paap (2009) and Wyse and Friel (2012), or of appropriately averaging over models, for example using reversible jump MCMC (Green, 1995).

Substantial developments in specialised methods for ordinal data have recently been made (see Liu & Agresti, 2005, for an overview). For instance, Fernández et al. (2014) have recently developed one- and two-dimensional clustering models for ordinal data having a likelihood-based foundation. They did this by using the assumption of the ordinal stereotype model, which allows the determination of a new spacing of the ordinal categories, as dictated by the data. The models presented in this paper may be extended to other ordinal models such as the adjacent-categories logit models, continuation-ratio logit models, and mean response models (see Agresti, 2012, for details on these models). Similarly, incorporating covariates into the model, when these are available, is straightforward by adjusting the linear predictor accordingly.

We have presented the case when *q*, i.e. the number of levels, is the same for all variables. However, the models are easily extended to allow for a set of cutpoints to be calculated for each unique value of *q* observed in the data set.

The area of application of these models is extremely wide and includes market research, where questions of the type “How likely are you to buy this product in the future” have possible responses “Very likely to buy”, “Likely to buy”, “May or may not buy”, etc. Additionally, the models are useful for services, such as websites, that review products, such as books, music albums, hotels. and provide recommendations to the users according to their own past reviews, as they can simultaneously cluster the individuals according to their taste, but also the products according to the reviews they have received from all users.

Future research will develop a graphical method for matrix visualisation, taking the resulting probabilities of allocation for each individual data point into account. The existing graphical methods rely on the use of ad hoc distance metrics and similarity measures which, as we have noted above, do not respect the full ordinal nature of the data.

## Electronic supplementary material

Below is the link to the electronic supplementary material.

Supplementary material 1 (pdf 103 KB)
