# Supplementary material for: Biclustering Models for Two-Mode Ordinal Data
Source: Psychometrika. 2016 Jun 21;81:611–24. doi: 10.1007/s11336-016-9503-3 (PMC4978779; doi:10.1007/s11336-016-9503-3)
Supplement: Supplementary file 1 — Supplementary material 1 (pdf 103 KB) [file 11336_2016_9503_MOESM1_ESM.pdf]

A. Details of the EM algorithm used to fit the proposed models

In this section we describe the model fitting procedure based on the EM-algorithm for the row clustering (Section A.1), column clustering (Section A.2), and biclustering (Section A.3) cases.

A.1. Row clustering

A.1.1. E-Step

The conditional expectation of the complete data log-likelihood at iteration t is

$$\begin{aligned}\ell_c(\Omega \mid \Omega^{(t-1)}) = &\sum_{i=1}^{n}\sum_{r=1}^{R} \log\left(\pi_r^{(t-1)}\right) E\left[Z_{ir} \mid \{y_{ij}\}, \Omega^{(t-1)}\right] \\ &+ \sum_{i=1}^{n}\sum_{j=1}^{p}\sum_{k=1}^{q}\sum_{r=1}^{R} I(y_{ij}=k) \log\left(\theta_{rjk}^{(t-1)}\right) E\left[Z_{ir} \mid \{y_{ij}\}, \Omega^{(t-1)}\right],\end{aligned} \tag{7}$$

where $\Omega = \{\boldsymbol{\phi}, \boldsymbol{\pi}\}$.

The latent variable Z_{ir} is a Bernoulli random variable so that

$$E\left[Z_{ir} \mid \{y_{ij}\}, \Omega^{(t-1)}\right] = P\left[Z_{ir}=1 \mid \{y_{ij}\}, \Omega^{(t-1)}\right] = \widehat{z}_{ir}^{(t)},$$

with

$$\widehat{z}_{ir}^{(t)} = \frac{P\left(\{y_{ij}\}, \Omega^{(t-1)} \mid Z_{ir}=1\right) P\left(Z_{ir}=1\right)}{\sum_{a=1}^{R} P\left(\{y_{ij}\}, \Omega^{(t-1)} \mid Z_{ia}=1\right) P\left(Z_{ia}=1\right)}$$
$$= \frac{\pi_r^{(t-1)} \prod_{j=1}^{p} \prod_{k=1}^{q} \left(\theta_{rjk}^{(t-1)}\right)^{I(y_{ij}=k)}}{\sum_{a=1}^{R} \left\{\pi_a^{(t-1)} \prod_{j=1}^{p} \prod_{k=1}^{q} \left(\theta_{ajk}^{(t-1)}\right)^{I(y_{ij}=k)}\right\}}. \tag{8}$$

Finally,

$$\ell_c(\Omega \mid \Omega^{(t-1)}) = \sum_{i=1}^{n} \sum_{r=1}^{R} \widehat{z}_{ir}^{(t)} \log\left(\pi_r^{(t-1)}\right) + \sum_{i=1}^{n} \sum_{j=1}^{p} \sum_{k=1}^{q} \sum_{r=1}^{R} \widehat{z}_{ir}^{(t)} I(y_{ij}=k) \log\left(\theta_{rjk}^{(t-1)}\right). \tag{9}$$

A.1.2. M-Step

The M-step has two parts:

1) Update the row cluster proportions using

$$\pi_r^{(t)} = \frac{1}{n} \sum_{i=1}^{n} E\left[Z_{ir} \mid \{y_{ij}\}, \Omega^{(t-1)}\right] = \frac{1}{n} \sum_{i=1}^{n} \widehat{z}_{ir}^{(t)}, \qquad r = 1, \ldots, R. \tag{10}$$

2) Update parameters in $\boldsymbol{\phi}$ by numerically maximizing the conditional expectation of the complete data log-likelihood shown in Equation (9).

A.2. Column clustering

A.2.1. E-Step

Now,

$$\widehat{x}_{jc}^{(t)} = \frac{\kappa_c^{(t-1)} \prod_{i=1}^{n} \prod_{k=1}^{q} \left(\theta_{ick}^{(t-1)}\right)^{I(y_{ij}=k)}}{\sum_{a=1}^{C} \left\{ \kappa_a^{(t-1)} \prod_{i=1}^{n} \prod_{k=1}^{q} \left(\theta_{iak}^{(t-1)}\right)^{I(y_{ij}=k)} \right\}} \tag{11}$$

and

$$\ell_c(\Omega \mid \Omega^{(t-1)}) = \sum_{j=1}^{p} \sum_{c=1}^{C} \widehat{x}_{jc}^{(t)} \log\left(\kappa_c^{(t-1)}\right) + \sum_{i=1}^{n} \sum_{j=1}^{p} \sum_{k=1}^{q} \sum_{c=1}^{C} \widehat{x}_{jc}^{(t)} I(y_{ij} = k) \log\left(\theta_{ick}^{(t-1)}\right), \tag{12}$$

where $\Omega = \{\boldsymbol{\phi}, \boldsymbol{\kappa}\}$.

A.2.2. M-Step

(1)

$$\widehat{\kappa}_c^{(t)} = \frac{1}{p} \sum_{j=1}^{p} \left(\frac{\kappa_c^{(t-1)} \prod_{i=1}^{n} \prod_{k=1}^{q} \left(\theta_{ick}^{(t-1)}\right)^{I(y_{ij}=k)}}{\sum_{a=1}^{C} \left\{ \kappa_a^{(t-1)} \prod_{i=1}^{n} \prod_{k=1}^{q} \left(\theta_{iak}^{(t-1)}\right)^{I(y_{ij}=k)} \right\}} \right)$$

and

(2) maximise the complete-data log-likelihood shown in Equation (12) with respect to parameters in $\boldsymbol{\phi}$.

A.3. Biclustering

A.3.1. E-Step

Following the same steps as in Sections A.1 and A.2, we obtain

$$\widehat{z}_{ir}^{(t)} = \frac{\pi_r^{(t-1)} \prod_{j=1}^{p} \prod_{k=1}^{q} \left\{ \sum_{c=1}^{C} \kappa_c \left(\theta_{rck}^{(t-1)} \right)^{I(y_{ij}=k)} \right\}}{\sum_{a=1}^{R} \pi_a^{(t-1)} \prod_{j=1}^{p} \prod_{k=1}^{q} \left\{ \sum_{c=1}^{C} \kappa_c \left(\theta_{ack}^{(t-1)} \right)^{I(y_{ij}=k)} \right\}} \tag{13}$$

and

$$\widehat{x}_{jc}^{(t)} = \frac{\kappa_c^{(t-1)} \prod_{i=1}^{n} \prod_{k=1}^{q} \left\{ \sum_{r=1}^{R} \pi_r \left(\theta_{rck}^{(t-1)} \right)^{I(y_{ij}=k)} \right\}}{\sum_{a=1}^{C} \kappa_a^{(t-1)} \prod_{i=1}^{n} \prod_{k=1}^{q} \left\{ \sum_{r=1}^{R} \pi_r \left(\theta_{rak}^{(t-1)} \right)^{I(y_{ij}=k)} \right\}}, \tag{14}$$

while

$$\begin{aligned} \ell_c(\Omega \mid \Omega^{(t-1)}) = & \sum_{i=1}^{n} \sum_{r=1}^{R} \log\left(\pi_r^{(t-1)}\right) E\left[Z_{ir} \mid \{y_{ij}\}, \Omega^{(t-1)}\right] \\ & + \sum_{j=1}^{p} \sum_{c=1}^{C} \log\left(\kappa_c^{(t-1)}\right) E\left[X_{jc} \mid \{y_{ij}\}, \Omega^{(t-1)}\right] \\ & + \sum_{i=1}^{n} \sum_{j=1}^{p} \sum_{k=1}^{q} \sum_{r=1}^{R} \sum_{c=1}^{C} I(y_{ij} = k) \log\left(\theta_{rck}^{(t-1)}\right) E\left[Z_{ir} X_{jc} \mid \{y_{ij}\}, \Omega^{(t-1)}\right], \end{aligned}$$

where $\Omega = \{\boldsymbol{\phi}, \boldsymbol{\pi}, \boldsymbol{\kappa}\}$.

There is a lack of a posteriori independence of $\{Z_{ir}\}$ and $\{X_{jc}\}$ which makes the evaluation of $E[Z_{ir}X_{jc}|\{y_{ij}\}, \Omega]$ in the E-step computationally

expensive as it requires a sum either over all possible allocations of rows to row groups, or over all possible allocations of columns to column groups. For that reason, the variational approximation of Govaert and Nadif (2005) is employed and we write

$$E\left[Z_{ir}X_{jc} \mid \{y_{ij}\},\Omega\right] \simeq E\left[Z_{ir} \mid \{y_{ij}\},\Omega\right] E\left[X_{jc} \mid \{y_{ij}\},\Omega\right] = \widehat{z}_{ir}\widehat{x}_{jc}.$$

Hence,

$$\begin{aligned}\ell_c(\Omega \mid \Omega^{(t-1)}) = &\sum_{i=1}^{n}\sum_{r=1}^{R}\widehat{z}_{ir}\log\left(\pi_r^{(t-1)}\right) + \sum_{j=1}^{p}\sum_{c=1}^{C}\widehat{x}_{jc}\log\left(\kappa_c^{(t-1)}\right)\\ &+\sum_{i=1}^{n}\sum_{j=1}^{p}\sum_{k=1}^{q}\sum_{r=1}^{R}\sum_{c=1}^{C}\widehat{z}_{ir}\widehat{x}_{jc}I(y_{ij}=k)\log\left(\theta_{rck}^{(t-1)}\right).\end{aligned} \tag{15}$$

A.3.2. M-Step

1)

$$\kappa_c^{(t)} = \frac{1}{p}\sum_{j=1}^{p}\left(\frac{\kappa_c^{(t-1)}\prod_{i=1}^{n}\prod_{k=1}^{q}\left(\theta_{ick}^{(t-1)}\right)^{I(y_{ij}=k)}}{\sum_{a=1}^{C}\left\{\kappa_a^{(t-1)}\prod_{i=1}^{n}\prod_{k=1}^{q}\left(\theta_{iak}^{(t-1)}\right)^{I(y_{ij}=k)}\right\}}\right)$$

and

$$\pi_r^{(t)} = \frac{1}{n}\sum_{i=1}^{n}\left(\frac{\pi_r^{(t-1)}\prod_{j=1}^{p}\prod_{k=1}^{q}\left(\theta_{rjk}^{(t-1)}\right)^{I(y_{ij}=k)}}{\sum_{a=1}^{R}\left\{\pi_a^{(t-1)}\prod_{j=1}^{p}\prod_{k=1}^{q}\left(\theta_{ajk}^{(t-1)}\right)^{I(y_{ij}=k)}\right\}}\right).$$

2) maximise the complete-data log-likelihood shown in Equation (15) with respect to parameters in $\boldsymbol{\phi}$.

B. Questionnaire considered in Section 4.1

1. What happens in my life is determined by God's purpose. (God 1)
2. My life is primarily controlled by God. (God 2)
3. When I am anxious, I rely on God for inner peace. (God 3)
4. Whether or not I get into a car accident depends on God's plans. (God 4)
5. In order to have my plans work, I make sure they fit in with the commands of God. (God 5)
6. When things don't go my way, I ought to pray. (God 6)
7. When faced with a difficult decision, I depend on God to guide my feelings and actions. (God 7)
8. When good things happen to me it is because of God's blessing. (God 8)
9. I feel like what happens in my life is mostly determined by powerful people. (External 1)
10. Although I might have good ability, I will not be given leadership responsibility without appealing to people in positions of power. (Ex-

ternal 2)

11. My life is chiefly controlled by people who are more powerful than me. (External 3)

12. People like myself have very little chance of protecting our personal interests when they conflict with those of strong pressure groups. (External 4)

13. Getting what I want requires pleasing those people above me. (External 5)

14. If important people were to decide they didn't like me, I probably wouldn't make many friends. (External 6)

15. Whether or not I get into a car accident depends mostly on the other driver. (External 7)

16. In order to have my plans work, I make sure they fit in with the desires of people who have power over me. (External 8)

C. Questionnaire considered in Section 4.2

1. (a) I do not feel sad.
   (b) I feel sad most of the time.
   (c) I am sad all the time.
   (d) I am so sad or unhappy that I can't stand it.

2. (a) I am not discouraged about my future.
   (b) I feel more discouraged about my future than I used to do.
   (c) I do not expect things to work out for me.
   (d) I feel my future is hopeless and will only get worse.

3. (a) I do not feel like a failure.
   (b) I have failed more than I should have.
   (c) As I look back, I see a lot of failures.
   (d) I feel I am a total failure as a person.

4. (a) I get as much pleasure as I ever did from the things I enjoy.
   (b) I don't enjoy things as much as I used to.
   (c) I get very little pleasure from the things I used to enjoy.
   (d) I am so sad or unhappy that I can't stand it.

5. (a) I don't feel particularly guilty.

   (b) I feel guilty over many things I have done or should have done.

   (c) I feel quite guilty most of the time

   (d) I feel guilty all of the time.

6. (a) I don't feel I am being punished.

   (b) I feel I may be punished.

   (c) I expect to be punished.

   (d) I feel I am being punished.

7. (a) I feel the same about myself as ever.

   (b) I have lost confidence in myself.

   (c) I am disappointed in myself.

   (d) I dislike myself.

8. (a) I don't criticise or blame myself more than usual .

   (b) I am more critical of myself than I used to be

   (c) I am sad all the time.

   (d) I am so sad or unhappy that I can't stand it.

9. (a) I don't have any thoughts of killing myself.

(b) I have thoughts of killing myself, but I would not carry them out.

(c) I would like to kill myself.

(d) I would kill myself if I had the chance.

10. (a) I don't cry more than I used to.

(b) I cry more than I used to.

(c) I cry over every little thing.

(d) I feel like crying, but I can't.

11. (a) I am no more irritable than usual.

(b) I am more irritable than usual.

(c) I am much more irritable than usual.

(d) I am irritable all the time.

12. (a) I have not lost interest in other people.

(b) I am less interested in other people than before.

(c) I have lost most of my interest in other people.

(d) I have lost all my interest in other people.

13. (a) I make decisions about as well as ever.

(b) I find it more difficult to make decisions than usual.

(c) I have much greater difficulty in making decisions than I used to.

(d) I have trouble making any decisions.
